# Bonus-freeze: benefit or risk? Two-year outcome and procedural comparison of a “bonus-freeze” and “no bonus-freeze” protocol using the second-generation cryoballoon for pulmonary vein isolation

**DOI:** 10.1007/s00392-016-0987-8

**Published:** 2016-04-16

**Authors:** Christian-H. Heeger, Erik Wissner, Peter Wohlmuth, Shibu Mathew, Kentaro Hayashi, Christian Sohns, Bruno Reißmann, Christine Lemes, Tilman Maurer, Ardan M. Saguner, Francesco Santoro, Johannes Riedl, Feifan Ouyang, Karl-Heinz Kuck, Andreas Metzner

**Affiliations:** Department of Cardiology, Asklepios Klinik St. Georg, Lohmühlenstr. 5, 20099 Hamburg, Germany

**Keywords:** Atrial fibrillation, Pulmonary vein isolation, Cryoballoon, Long-term follow-up

## Abstract

**Background:**

Second-generation cryoballoon based pulmonary vein isolation has demonstrated encouraging acute and mid-term clinical outcome. Customarily, a bonus-freeze is applied after successful pulmonary vein isolation.

**Objective:**

To compare the long-term clinical outcome and safety profile of a bonus-freeze and a no bonus-freeze protocol.

**Methods:**

A total of 120 consecutive patients with paroxysmal [95/120 (79 %)] or persistent atrial fibrillation [25/120 (21 %)] underwent CB2-based PVI. Freeze-cycle duration was 240 s. In the first 60 patients a bonus-freeze was applied after successful PVI (group 1), while in the following 60 patients the bonus-freeze was omitted (group 2).

**Results:**

Procedure and fluoroscopy times were significantly shorter in group 2 [113.8 ± 32 vs 138.2 ± 29 min (*p* = 0.03) and 19.2 ± 6 vs 24.3 ± 8 min (*p* = 0.02)]. No differences in procedural complications were found. During a mean follow-up of 849 ± 74 (group 1) and 848 ± 101 days (group 2, *p* = 0.13) 69 % of patients (group 1) and 67 % of patients (group 2) remained in stable sinus rhythm without any differences between the groups (*p* = 0.69).

**Conclusions:**

Freedom from atrial fibrillation after second-generation cryoballoon based pulmonary vein isolation and a follow-up of >2 years is comparable when applying a bonus- and a no bonus-freeze protocol, while procedure and fluoroscopy times are significantly shorter when omitting the bonus-freeze. No differences in periprocedural complications were identified.

## Introduction

Second-generation cryoballoon (CB2, Arctic Front Advance, Medtronic, Inc., Minneapolis, MN, USA)-based pulmonary vein isolation (PVI) has demonstrated high procedural success rates and encouraging clinical outcome data for patients with paroxysmal (PAF) and persistent atrial fibrillation (PersAF) [1–7]. Current ablation strategies are commonly based on a fixed freeze-cycle duration of 180–240 s followed by a bonus-freeze-cycle of the same duration following successful PVI [1, 3, 5, 8]. However, collateral damage to extra cardiac structures such as phrenic nerve palsy (PNP) and esophageal thermal injury is related to minimal balloon and esophageal temperatures as well as to freeze-cycle durations and freeze-cycle numbers [9, 10]. To prevent damage to extra cardiac structures, shorter freeze-cycle durations and omitting the bonus-freeze-cycle were suggested [2, 4]. However, no direct comparison with regard to long-term clinical outcome and the safety profile of bonus-freeze and no bonus-freeze protocols have been performed yet. Therefore, the impact of the bonus-freeze-cycle remains to be evaluated. The current study investigates the long-term clinical outcome following CB2-based PVI applying two different ablation strategies: bonus-freeze and no bonus-freeze application following successful PVI and evaluates the safety profile of both ablation strategies.

## Methods

### Patient characteristics and study design

Consecutive patients with symptomatic, drug-refractory PAF or short-standing PersAF (duration of ≤3 months) were admitted and consented for CB2-based PVI. Exclusion criteria were prior left atrial (LA) ablation, LA diameter >60 mm, severe valvular heart disease or contraindications to postinterventional oral anticoagulation. Transesophageal echocardiography was performed prior to ablation in all patients to assess the LA diameter and to rule out intracardiac thrombi. No additional preprocedural imaging was performed. All patients gave written informed consent and all patient information was anonymized. The study was approved by the local ethic’s board and has been performed in accordance with the ethical standards laid down in the 1964 Declaration of Helsinki and its later amendments.

### Intraprocedural management

All procedures were performed under deep sedation using midazolam, fentanyl, and propofol. Prior to transseptal puncture, two diagnostic catheters were introduced via the right femoral vein and positioned within the coronary sinus and along the His-bundle. Single transseptal puncture was performed under fluoroscopic guidance using a modified Brockenbrough technique and an 8.5F transseptal sheath (SL1, St. Jude Medical, Inc., St. Paul, MN, USA). The transseptal sheath was exchanged over a wire for a 12F steerable sheath (Flexcath Advance, Medtronic, Inc., Minneapolis, MN, USA). Heparin bolus were administered targeting an activated clotting time of >300 s. Subsequently, selective PV angiographies were performed to identify the individual PV ostia. A temperature probe (Sensitherm, St. Jude Medical) was placed within the esophagus at the level of the individual CB2 position to monitor esophageal temperatures during the freeze-cycles. The intraluminal esophageal temperature cut-off was set at 15 °C [2].

### PVI using the second-generation 28 mm CB

The CB2 was advanced into the LA over a modified spiral mapping catheter (15 or 20 mm diameter; Achieve^TM^, Medtronic, Inc., Minneapolis, MN, USA). The CB2 was inflated proximal to the PV ostium followed by gentle push aiming for complete sealing at the antral aspect of the PV. Contrast medium injected through the central lumen of the CB2 was used to verify complete PV occlusion. This was followed by a freeze-cycle of 240 s. After successful PVI one additional bonus-freeze-cycle of 240 ms duration was applied in the first 60 patients, while the bonus-freeze-cycle was omitted in the following 60 patients. Prior to each CB2 application a selective PV angiography was performed to ensure the correct CB2 position at the PV ostium and complete PV occlusion. Electrical isolation of the PVs was always performed in the same order: left superior PV (LSPV) followed by the left inferior PV (LIPV), right superior PV (RSPV) and the right inferior PV (RIPV). The procedural endpoint was defined as persistent PVI verified by spiral mapping catheter recordings 30 min after the last energy application.

### Phrenic nerve pacing

During CB2 application along the septal PVs continuous pacing of the phrenic nerve was performed using a diagnostic catheter positioned within the superior vena cava (7F, Webster TM, Biosense Webster, Inc.) [2]. Pacing was set at maximum output and pulse width (12 mA, 2.9 ms) and a cycle length of 1200 ms. Phrenic nerve capture was monitored by tactile feedback of diaphragmatic contraction and assessment of the right diaphragmatic compound motor action potential (CMAP) [11, 12]. Energy delivery was interrupted immediately if weakening or loss of diaphragmatic contraction was noted or a decrease of the CMAP amplitude of ≥30 % was observed. In case of catheter dislodgement, the pacing catheter was repositioned until phrenic nerve capture was re-obtained. In case of persistent PNP, no further cryoenergy was delivered along the septal PVs.

### Postprocedural care

Following PVI, all patients underwent transthoracic echocardiography to rule out pericardial effusion. All patients were treated with proton-pump inhibitors twice daily for 6 weeks. Low molecular-weight heparin was administered in patients on vitamin K antagonists and an INR <2.0 until a therapeutic INR of 2–3 was achieved. Novel oral anticoagulants were reinitiated 6 h post ablation. Anticoagulation was continued for at least 3 months and thereafter based on the individual CHA_2_DS_2_-VASC-score. Previously ineffective antiarrhythmic drugs were continued for 3 months.

### Repeat procedures

In patients admitted for a repeat procedure due to recurrence of atrial arrhythmia, venous access and transseptal puncture were performed as previously described. The presence or absence of electrical activation of the PVs was assessed using a spiral mapping catheter. An electroanatomical LA map (Carto^TM^, Biosense Webster) was generated and the PV ostia were tagged. Identified reconduction gaps were closed by irrigated radiofrequency (RF) ablation using a 3.5 mm irrigated-tip catheter (Biosense Webster, Navi-Star^TM^, Thermo-Cool^TM^). The procedural endpoint was complete electrical PVI [13]. In patients with persistent isolation of all PVs admitted in SR, fractionated ostial potentials along previously performed ablation lines were identified and ablated and/or linear lesion sets were applied. In patients admitted in AF or atrial tachycardia (AT) and persistent isolation of all PVs, ostial potentials were identified and ablated followed by ablation of complex fractionated atrial electrograms and deployment of linear lesion sets in case of conversion to an AT [13].

### Follow-up

Patients completed outpatient clinic visits at 3, 6, 12 months and in 6-months intervals thereafter including 12-lead surface ECG and 24 h-Holter ECGs. In addition, regular telephonic interviews were performed. Additional outpatient clinic visits were immediately initiated in case of symptoms suggestive for recurrent arrhythmia [1–3]. The primary endpoint was defined as recurrence of any symptomatic or documented atrial arrhythmia >30 s following a blanking period of 3 months. Secondary endpoints were defined as procedure related complications such as PNP, cerebral embolism or atrioesophageal fistula.

### Statistical analysis

Differences of metric variables between the two groups were analyzed with *t* test if the data were normally distributed, and with Wilcoxon–Mann–Whitney test otherwise. Differences between categorical variables were evaluated using the Chi square test or the Fisher’s exact test in case of small expected cell frequencies. PV related data were analyzed with (generalized) linear mixed models. For patient related data the Wilcoxon test (contrast) and Fisher’s exact tests (phrenic nerve palsy and pericardial effusion) were used. Linear mixed models were used for continuous data. Generalized linear mixed models were applied for binary or count data. A hierarchical logistic regression model was consulted for binary data. A poisson distribution was assumed for count data. All *p* values are two-sided and a *p* value < 0.05 was considered significant. All calculations were performed with the statistical analysis software SAS (SAS Institute Inc., version 9.3, Cary, NC, USA) [1–3].

## Results

### Patient characteristics

**Fig. 1:**
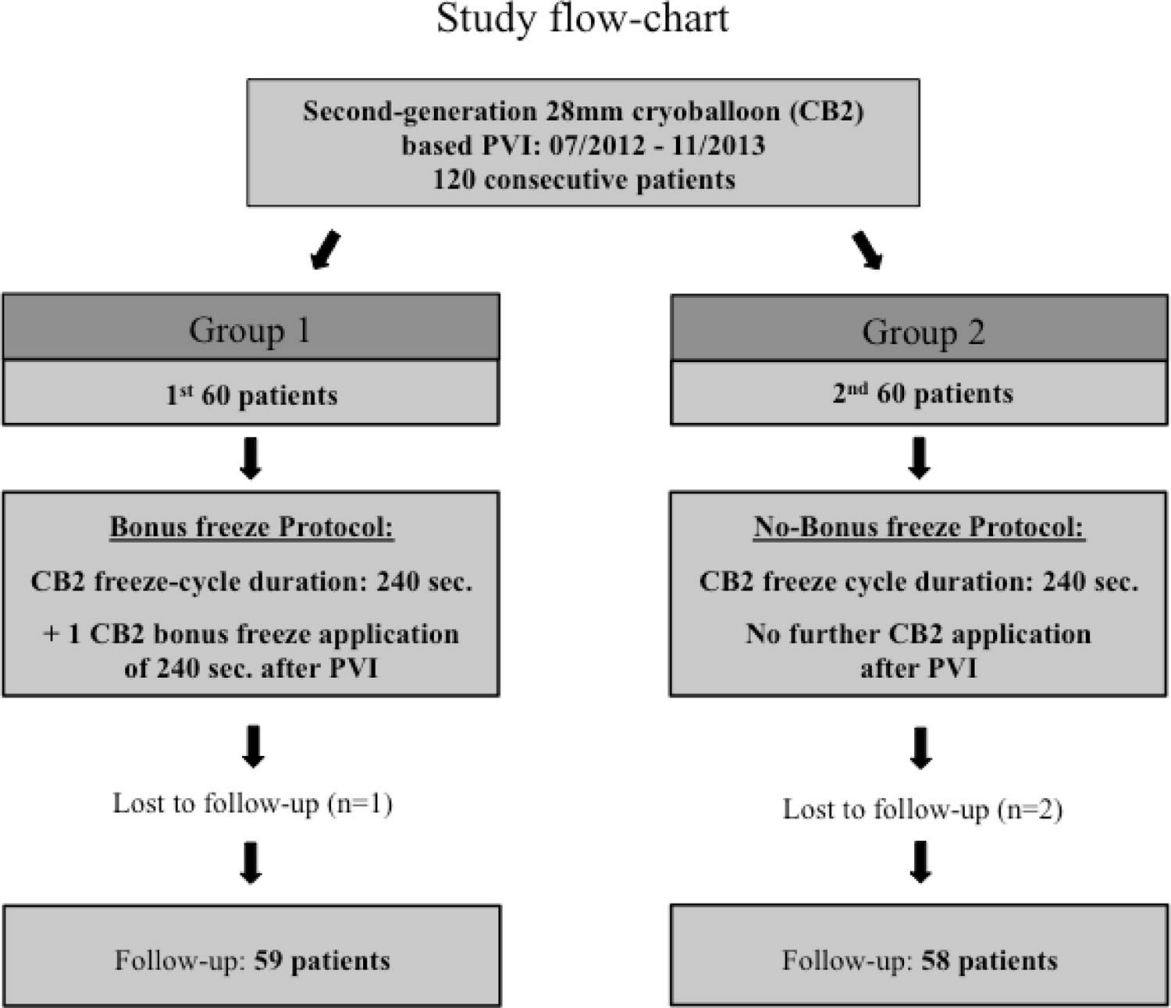
Study flow chart. *CB2* second-generation 28 mm cryoballoon, *PVI* pulmonary vein isolation

A total of 120 patients with PAF [95/120 (79 %)] or short-standing (<3 months duration) PersAF [25/120 (21 %)] underwent CB2-based PVI. No differences in baseline characteristics were found between the groups (Table 1). In the first 60 patients a bonus-freeze-cycle was applied following successful PVI (group 1) while in the following 60 patients the bonus-freeze-cycle was omitted (group 2) (Fig. 1).

**Table 1:** Baseline characteristics

	Group I (bonus-freeze)	Group II (no bonus-freeze)	*p*
Patients, *n* Age (years)	60 62 ± 11	60 61 ± 11	0.84
Female gender, *n* (%)	24 (40)	22 (37)	0.24
Paroxysmal AF, *n* (%)	45 (75)	50 (83)	0.54
Short persistent AF, *n* (%)	15 (25)	10 (17)	0.54
Duration of AF (years)	36 ± 40	35 ± 31	0.31
LA-size (mm)	43 ± 5	42 ± 8	0.27
Arterial hypertension, *n* (%)	42 (70)	37 (62)	0.86
Diabetes mellitus, *n* (%)	8 (13)	5 (8)	0.36
Coronary artery disease, *n* (%)	6 (10)	9 (15)	0.10
Prior stroke, *n* (%)	3 (5)	5 (8)	0.11
Mean CHA_2_DS_2_-VASc-score	1.95	1.8	0.27

*AF* atrial fibrillation, *LA* left atrium

### Acute ablation results

In patients of group 1 231 PVs were identified [60 RSPVs, 60 RIPVs, 57 LSPVs, 57 LIPVs and 3 left common PVs (LCPV)] (Table 2). A total of 230/231 (99 %) PVs were successfully isolated. One RIPV was not targeted due to PNP during CB2 ablation along the RSPV. In patients of group 2 237 PVs were identified (60 RSPVs, 60 RIPVs, 51 LSPVs, 51 LIPVs and 9 LCPVs) and a total of 235/237 (99 %) PVs were successfully isolated. Two RIPV were not targeted due to PNP during CB2 application of the ipsilateral RSPV. The median (1st, 3rd quartile) number of total freeze-cycles was 2 (2, 2) for group 1 and 1 (1, 1) for group 2 (*p* < 0.001), while the median number of freeze-cycles to PVI was equal for both groups 1 (1, 1) group 1 and 1 (1, 1) for group 2 (*p* = 0.24). The minimal ballon temperature was found to be significantly different with −51.1 ± 6 °C (group 1) and −48.2 ± 6 °C (group 2), (*p* < 0.001). Real-time PVI was visualized in 102/230 (44 %) and 102/235 (43 %) of targeted PVs in patients of group 1 and group 2, respectively (*p* = 0.85). No differences were found for the median (1st, 3rd quartile) time to PVI between the two groups (group 1 = 40 (27, 65 s) and group 2 38 (28, 55), *p* = 0.32.

**Table 2:** Comparison of procedural data

	Group I (bonus-freeze)	Group II (no bonus-freeze)	*p*
Number of PVs, *n*	231	237	
Total CB cycles per PV	2 (2, 2)	1 (1, 1)	<0.001
Total CB cycles per PV until PVI	1 (1, 1)	1 (1, 1)	0.24
Number of isolated PVs, *n* (%)	230/231 (99)	235/237 (99)	0.66
Minimal CB2 temperature (°C)	−51.1 ± 6	−48.2 ± 6	<0.001
Minimal esophageal temp. (°C)	34.6 (31, 36)	35.3 (34, 36)	0.05
Time to PVI (s)	40 (27, 65)	38 (28, 55)	0.32
Procedure time (min)	138.2 ± 29	113.8 ± 32	0.03
Fluoroscopy time (min)	24.3 ± 8	19.2 ± 6	0.02
Amount of contrast medium (ml)	160 (150, 200)	120 (100, 140)	<0.001
Phrenic nerve palsy, *n* (%)	2 (3)	3 (5)	1.00

Values are expressed as mean and SD if data were normally distributed or as median (1st, 3rd quartile)

*PV(s)* Pulmonary vein(s), *CB2* second-generation 28 mm cryoballoon, *PVI* pulmonary vein isolation

The mean procedure time was 138.2 ± 29 min (group 1) and 113.8 ± 32 min (group 2), (*p* = 0.03), while fluoroscopy time was 24.3 ± 8 min (group 1) and 19.2 ± 6 min (group 2), (*p* = 0.03). The amount of injected contrast medium was 160 ml (150, 200) and 120 ml (100, 140), respectively (*p* < 0.001).

### Complications

PNP occured in 2/60 (3 %) patients of group 1 and 3/60 (5 %) of group 2 (*p* = 1.00). Two PNP recovered within 10 months post ablation, another 2 PNP within 12 months and 1 PNP had fully recovered after 18 months. In 1/60 (1.7 %) patient of group 2 a pericardial tamponade occured following isolation all PVs most likely due to a difficult transseptal puncture. Pericardiocentesis was performed and the patient was discharged 2 days post ablation. No symptomatic PV stenosis, cerebral embolism, or atrioesophageal fistula occurred in any patient.

### Clinical follow-up

**Fig. 2:**
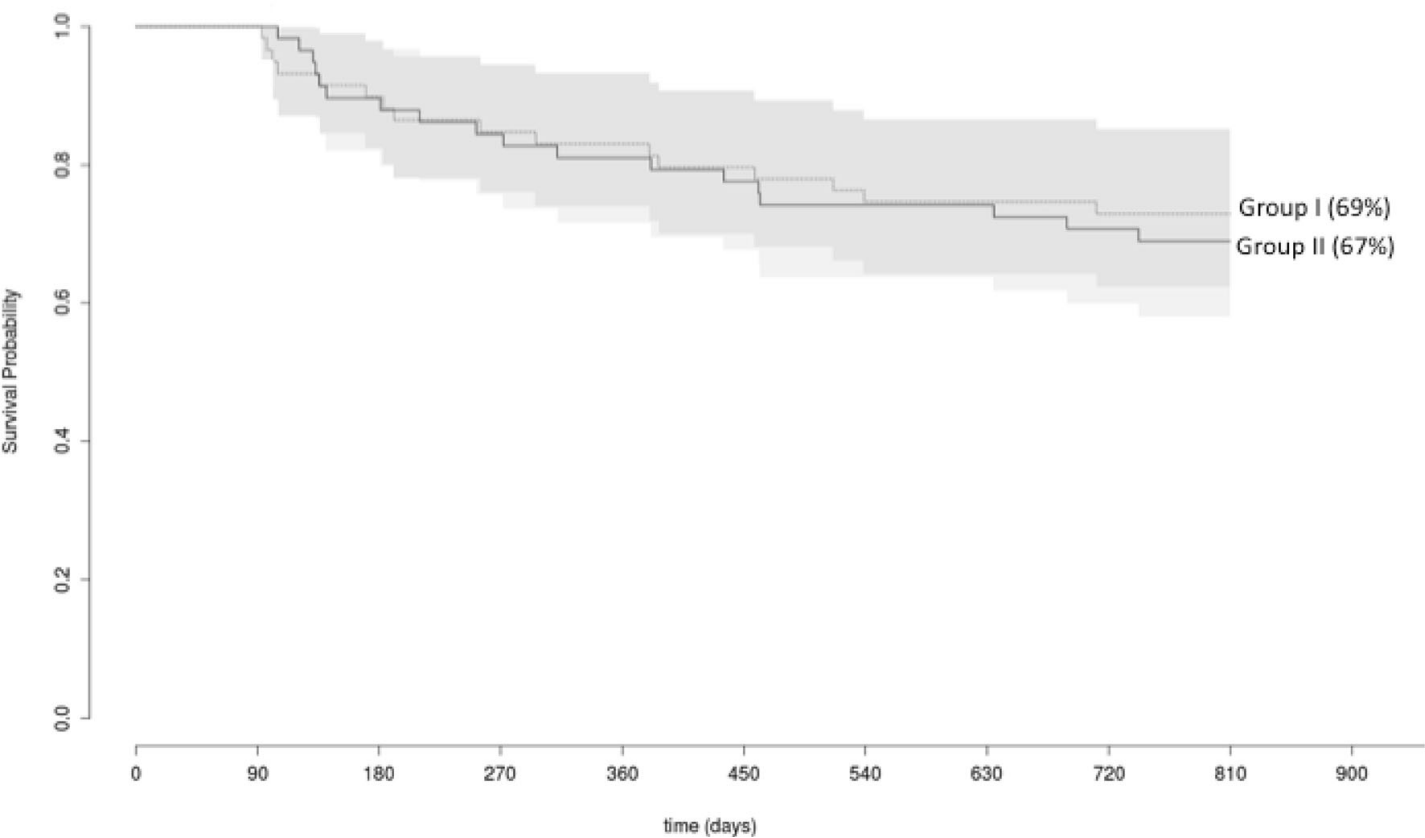
The Kaplan–Meier curve demonstrates the relative proportion of patients in stable sinus rhythm following index pulmonary vein isolation using the second-generation 28 mm cryoballoon during a follow-up period of 849 ± 74 (group 1) and 848 ± 101 days (group 2, *p* = 0.13). 69 % of patients (group 1) and 67 % of patients (group 2) remained in stable sinus rhythm during the follow-up period (*p* = 0.69)

Clinical follow-up was obtained in 117/120 (98 %) patients, while 3/120 (2 %) patients were lost to follow-up (1 patient of group 1, 2 patients of group 2). Mean follow-up duration was 849 ± 74 (group 1) and 848 ± 101 days (group 2, *p* = 0.13), respectively. A total of 42/59 (69 %) patients (group 1) and 41/58 (67 %) patients (group 2) remained in stable sinus rhythm during follow-up (*p* = 0.69). Eighteen out of 59 (31 %) patients of group 1 suffered from recurrence of atrial arrhythmias: 8/18 (44 %) patients PAF, 7/18 (39 %) patients PersAF and 3/18 (17 %) patients AT. Nineteen out of 58 (33 %) patients of group 2 presented with atrial arrhythmia recurrences 7/19 (37 %) patients PAF, 9/19 (47 %) patients PersAF and 3/19 (16 %) patients AT (Fig. 2).

### Findings during re-ablation procedures

**Fig. 3:**
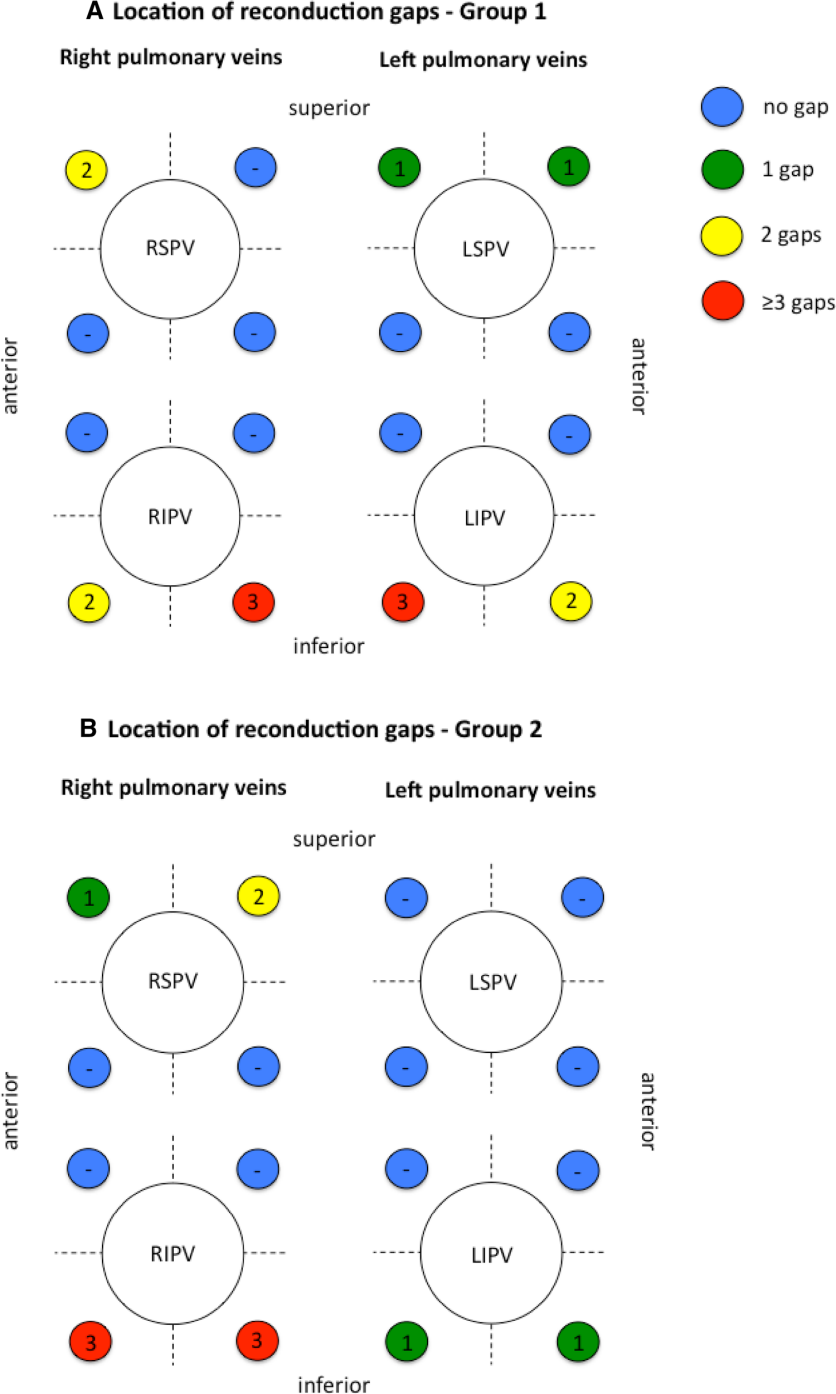
Location of electrical reconduction gaps. The figure depicts the number and location of reconduction gaps identified during re-ablation procedures. **a** Findings for group 1 (bonus-freeze protocol), **b** findings for group 2 (no bonus-freeze protocol). Septal and lateral pulmonary vein ostia are divided into four segments (antero-superior, antero-inferior, postero-superior, postero-inferior). *Numbers* express reconduction *gaps* found for each segment. *No gaps* were found along the carina between the ipsilateral pulmonary veins. Data for a *single left* common pulmonary vein is not shown (each group *n* = 1 single left common pulmonary vein, each with one gap. *RSPV* right superior pulmonary vein, *RIPV* right inferior pulmonary vein, *LSPV* left superior pulmonary vein, *LIPV* left inferior pulmonary vein)

A total of 26/34 (76 %) patients suffering from atrial arrhythmia recurrences underwent a second ablation procedure using RF energy (group 1: 12/18, 67 % and group 2: 14/19, 74 %). Procedural data of the second ablation attempt was available in 11/12 (92 %) patients of group 1 and 14/14 (100 %) patients of group 2. In a total of 27/98 (28 %) PVs, LA-to-PV reconduction was demonstrated. The LA-to-PV reconduction rate was comparable for patients of group 1 (15/43 PVs, 35 %) and group 2 (12/55 PVs, 22 %) (*p* = 0.16). In total, 27 reconduction gaps were identified (group 1: *n* = 15, group 2: *n* = 12) and distributed as shown in Fig. 3. Of note, no gap was found along the inter-PV section. Re-isolation of all PVs was successfully performed using RF energy. In 17/25 (68 %) patients [group 1: 7/11 (64 %), group 2: 10/14 (71 %)], ablation of PV reconduction gaps was conducted. In addition, ablation of ostial potentials was performed in 13/25 (52 %) patients [group 1: 2/11 (18 %), group 2: 3/14 (21 %)], ablation of complex fractionated atrial electrograms in 3/25 (12 %) patients [group 1: 1/11 (9 %), group 2: 2/14 (14 %)], and linear lesion ablation in 6/25 (24 %) patients. A mitral isthmus line ablation was performed in 1/11 (9 %) patients of group 1 and 2/14 (14 %) patients of group 2 while ablation of an anterior line was conducted in 1/11 (9 %) patients of group 1 and 2/14 (14 %) patients of group 2. Ablation of the cavo-tricuspid isthmus (CTI) was conducted in 6/25 (24 %) patients [group 1: 3/11 (27 %), group 2: 3/14 (21 %)] due to documented typical CTI-dependent atrial flutter. Periprocedural stroke occurred in 1/25 (4 %) patient of group 1. No further complications were documented.

## Discussion

To the best of our knowledge, the current study is the first to compare long-term clinical outcome and procedural characteristics of a bonus-freeze protocol and a no bonus-freeze protocol in CB2-based PVI. The study could demonstrate that omitting the bonus-freeze-cycle results in comparable clinical outcome data and significantly shorter procedure and fluoroscopy times without differences in the safety profile.

Current CB2 ablation strategies are mainly based on fixed freeze-cycle durations and mostly include a customized bonus-freeze-cycle following successful PVI [1, 5, 14]. However, recent publications suggest that omitting the bonus-freeze-cycle after successful PVI might be equally effective with regard to clinical outcome [2, 4]. While one-year clinical outcome in protocols that include a bonus-freeze-cycle range between 76.8 and 83.6 %, [1, 5, 8] slendered protocols without a bonus-freeze-cycle also report on 80.4 to 82 % 1-year clinical efficacy [2, 4]. Furthermore no differences were reported for durability PVI comparing a bonus- and a no bonus-freeze protocol in repeat procedures [13]. Additionally, our recent work demonstrated this observation in a smaller patients population. However, except a single study with limited follow-up duration, there are currently no publications in a head-to-head fashion comparing both strategies with regard to long-term clinical outcome [15].

Reducing the number of freeze-cycles and/or the freeze-cycle durations might not only affect clinical outcome of CB2 ablation but might also have a beneficial impact on its safety profile. Characteristic complications in CB2 ablation are PNP [9, 11, 16, 17] and thermal oesophageal lesions [18, 19]. Reduction in energy transfer might reduce the incidence of these complications and will simultaneously shorten procedure and fluoroscopy times and reduce radiation exposure. In the current study we could demonstrate significantly shorter procedure and fluoroscopy times as well as lower amounts of applied contrast media in the no bonus-freeze group, while long-term clinical outcome data was similar in both groups. Accordingly, our data support the hypothesis that the necessity of a bonus-freeze application after successful PVI is questionable. Although our study did not reveal a significant difference in periprocedural complications when omitting the bonus-freeze-cycle, it also failed to prove potential benefits of a bonus-freeze-cycle strategy with regard to clinical outcome.

Further studies will also have to evaluate and potentially include the individual time to isolation (TTI) as revealed by the Achieve-catheter. In some studies an increased incidence of online PV recordings was demonstrated when comparing the first-generation cryoballoon (Arctic Front, Medtronic) with the CB2 [20, 21]. In a recent study we could additionally demonstrate a significantly increased incidence of online PV recordings when using the recently launched third-generation CB (Arctic Front Advance ST, Medtronic) as compared to the CB2, thus facilitating the consideration of the individual TTI [22]. Future studies will focus on the TTI and thus on ablation strategies with individualized freeze-cycle durations considering the TTI. These potentially shorter freeze-cycles might further affect the safety profile but will also have to demonstrate equal clinical efficacy.

## Limitations

The presented findings are based on a single-center experience enrolling only a limited number of patients in a non-randomized fashion. However, the baseline characteristics in the current study are not different between the groups. The data is based on a retrospective analysis and the follow-up was limited to 24 h-Holter ECGs. This limitation might overestimate the overall success rate after CB2 ablation. Furthermore, the rate of complications utilizing the CB2 is considerably low in both groups. Therefore, higher patient numbers will be necessary to detect differences in periprocedural complications when applying a bonus-freeze versus a no bonus-freeze ablation protocol.

## Conclusions

The two-year freedom from atrial arrhythmia recurrences after CB2-based PVI is 69 % for the bonus-freeze and 67 % for the no bonus-freeze protocol. Procedure and fluoroscopy times, radiation exposure and the amount of applied contrast medium are significantly reduced when omitting the bonus-freeze application. No differences in periprocedural complications were detected. These findings suggest that a bonus-freeze application may not be essential for CB2 based PVI procedures.
